# Can near-peer medical students effectively teach a new curriculum in physical examination?

**DOI:** 10.1186/1472-6920-13-165

**Published:** 2013-12-11

**Authors:** Wolfgang A Blank, Hannes Blankenfeld, Roger Vogelmann, Klaus Linde, Antonius Schneider

**Affiliations:** 1 Institute of General Practice, Technische Universität München, Orleansstr. 47, 81667 Munich, Germany; 2 Medizinische Klink II, Technische Universität München, Munich, Germany; 3 Medizinische Klinik II, Universitätsklinikum Mannheim, Mannheim, Germany

## Abstract

**Background:**

Students in German medical schools frequently complain that the subject ‘clinical examination’ is not taught in a satisfying manner due to time constraints and lack of personnel resources. While the effectiveness and efficiency of practice-oriented teaching in small groups using near-peer teaching has been shown, it is rarely used in German medical schools. We investigated whether adding a new near-peer teaching course developed with student input plus patient examination under supervision in small groups improves basic clinical examination skills in third year medical students compared to a traditional clinical examination course alone.

**Methods:**

Third year medical students registered for the mandatory curricular clinical examination course at the medical faculty of the Technische Universität München were invited to participate in a randomised trial with blinded outcome assessment. Students were randomised to the control group participating in the established curricular physical examination course or to the intervention group, which received additional near-peer teaching for the same content. The learning success was verified by a voluntary objective structured clinical examination (OSCE).

**Results:**

A total of 84 students were randomised and 53 (63%) participated in the final OSCE. Students in the control group scored a median of 57% (25th percentile 47%, 75th percentile 61%) of the maximum possible total points of the OSCE compared to 77% (73%, 80%; p < 0.001) for students in the intervention group. Only two students in the intervention group received a lower score than the best student in the control group.

**Conclusion:**

Adding a near-peer teaching course to the routine course significantly improved the clinical examination skills of medical students in an efficient manner in the context of a resource-constrained setting.

## Background

Students in German medical schools frequently complain about the quality of the teaching of the course clinical examination [1]. Although physicians are generally enthusiastic about teaching, contractual obligations lead to time pressures [2] which may occur because of several reasons. With the increasing demands of the economy, even university hospitals are under economic pressure to treat more patients in a shorter time. Thus teaching time is even more limited than in the past. Additionally, these financial constraints lead to patients being discharged earlier making it difficult for students to find willing patients with suitable health conditions. Less time and fewer adequate patients lead to larger teaching groups, changing the method of teaching-away from bedside teaching on the wards back to the lecture room [3]. In addition, not all clinicians are enthusiastic about teaching clinical skills in the medical ward [4]. If there is a lack of clear tutorial objectives, teaching methods and content may vary widely between physicians which, in many cases, leads to a lack of hands-on experience for the student, resulting in insufficient skills in clinical examination [4].

Peer and near-peer teaching is one approach to deal with many of these problems. It reduces teaching demands on faculty and has been shown to sustain quality teaching in resource-constrained settings [5,6]. In the musculoskeletal clinical examination, the effectiveness of trained near-peer teachers in hands-on small-group practice was shown [7]. This was also demonstrated for medical wards [8]. Furthermore, additional benefits are that the near-peer tutors teach at the cognitive level of the students, practise peer feedback and develop leadership and teaching skills while the students being taught, learn in a safe and comfortable environment [6]. There is empirical evidence that trained students in the advanced phase of medical school are as good as experienced clinicians for teaching physical examination to younger peers [4,7]. If properly supervised, younger students accepted and were satisfied by such near-peer teaching [5,7].

Although the effectiveness and efficiency of near-peer teaching has been shown [9], it is rarely implemented in German medical faculties [1]. We investigated whether adding a new near-peer teaching course, based on a new curriculum developed with student input and incorporating patient examination under supervision in small groups, improves basic clinical examination skills in third year medical students compared to a traditional clinical examination course alone.

## Methods

### Study design and participants

The study was designed as a prospective randomised trial with blinded outcome assessment. All participants were third year students of the Medical School of the Technische Universität München (Munich, Germany). All students attending the mandatory physical examination course in the academic half-year 2009/2010 were eligible for the study. Students were informed about the study via the university intranet and during the first lecture at the beginning of the year. They were invited to an introductory meeting and given further information about the study. One hundred and eight students attended this meeting. Students were randomised to the control group, participating only in the current physical examination course, or to the intervention group, which received the new course in addition. The random sequence was computer generated and allocation fully concealed (gender-stratified allocation of random numbers in the complete list of participants in MS Excel). Both groups were evaluated by objective structured clinical examination (OSCE) two months after completion of the training. Students randomised to the control group were offered to participate in the near-peer teaching course in the following academic half-year. The ethics committee of the Medical Faculty of the Technische Universität München approved the study.

### Current physical examination course

At the time the study was performed, the mandatory physical examination course consisted of a theoretical and a practical part. The theoretical part comprised of eleven lectures held in a lecture theatre (three on history taking, eight on clinical examination skills; 45 minutes each). Additionally, students had to participate in 14 corresponding tutorials in groups of 20 in the wards of the teaching hospital. Each tutorial lasted 90 minutes and included a demonstration of examination techniques by an experienced physician and the opportunity to learn the skills in groups by peer-examination. Some students also had the opportunity to examine real patients in this context, depending on the ward and the trainer. Eight tutorials focussed on general physical examination skills, while six tutorials (two each) were on ENT (ear, nose, throat), ophthalmology and neurology. There was no structured curriculum for these tutorials and anecdotal evidence suggested that the content, method and quality varied considerably.

### Additional near-peer teaching course

Physicians of the relevant disciplines responsible for the routine clinical examination course, in collaboration with advanced medical students, developed a teaching concept for basic physical examination skills based on the near-peer teaching approach (Table 1). Voluntary near-peer tutors were recruited from fourth-or fifth-year students. Based on a newly developed course guide, structured according to the content of the curricular physical examination course, near-peer tutors were trained and finally accredited by the responsible coordinating physician.

**Table 1:** Didactic methods used in this study

**Activity**	**Current curriculum**	**Intervention group**
Lectures	**X**	**X**
Ward based tutorials (large groups)	**X**	
Demonstration workshop		**X**
Small group teaching		**X**
Peer physical examination under supervision		**X**
Supervised patient examination		**X**

The near-peer teaching course started with an interactive physician-led tutorial. Five physicians responsible for the curricular examination course demonstrated examination techniques to groups of nine students who rotated around five topic-related work stations (ophthalmology, ENT, abdomen, thorax and neurology, 25 minutes each). Afterwards students met for weekly tutorials supervised by near-peer tutors in small groups of three. For each topic, students completed three small group tutorials. First, students examined each other supervised by a tutor, next they examined each other on their own and finally a patient was examined under supervision of a near-peer tutor. The patients who were examined had been screened to ensure that they did not have any severe pathological findings within the focus of clinical examination to make sure that students first learn to recognize normal findings (e.g. abdominal examination in ophthalmological patients, ENT examination in patients after knee surgery). Experienced and trained near-peer tutors continuously supervised peer- and patient examinations. Every near-peer tutor taught the same examination throughout the course. The coordinating physicians were at the tutor’s disposal in case of questions or issues with patient recruitment.

### Evaluation

Students from both groups were invited to participate in an OSCE two months after the training. The OSCE contained clinical skill stations based on the curricular physical examination course. The near-peer tutors who did the teaching were not aware of the details of the OSCE.

The medical students had to demonstrate examination techniques using standardized patient actors. Trained raters, final year students not involved in the teaching course, were blinded with respect to randomised groups. Raters used evaluation sheets to indicate if the examinee performed the examination as required. Points were awarded for correct execution of each part of the examination technique. At each of the five stations, a maximum of 40 points (36 points on technical skills and 4 points on communication skills) were obtainable, totalling a maximum score of 200 points (main outcome measure). Marks were assigned according to usual German standard procedures (> 74% of maximum points = very good, 63-74% = good, 54-62% = satisfactory, 38-53% = sufficient, < 38% insufficient).

Students, tutors and clinical teachers gave feedback at the end of the course in a final meeting.

### Statistical analysis

Data were entered into a SPSS database. OSCE scores were transformed into percentages of maximum points. For all students participating in the OSCE, data were complete. Data for students not attending the OSCE were not analysed. Analyses were done using IBM SPSS 19. Medians, quartiles, means, standard deviations (SDs), frequency counts and proportions were used to summarize data descriptively. As the distribution was asymmetrical for some of the OSCE scales we used the Mann-Whitney-U-Test to test for differences between groups.

## Results

**Figure 1:**
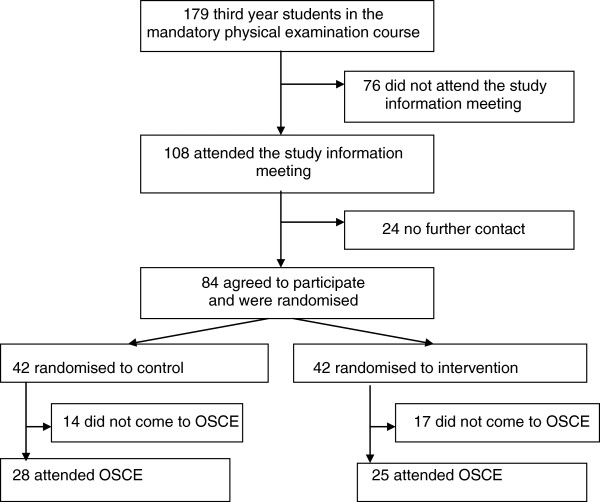
Trial flow chart.

84 of 179 eligible students agreed to participate and were randomly assigned to control (n = 42, 29 female, mean age of 22.9 (SD 2.9) years) or intervention group (n = 42, 30 female, mean age 23.6 (SD 3.1) years). 31 students did not attend the OSCE leaving 53 (63% of those randomised; see Figure 1) for final analysis: 28 (67%) students of the control group (21 female, 23.2 (SD 2.2) years) and 25 (59%) students of the intervention group (17 female, 22.2 (SD 0.9) years).

**Figure 2:**
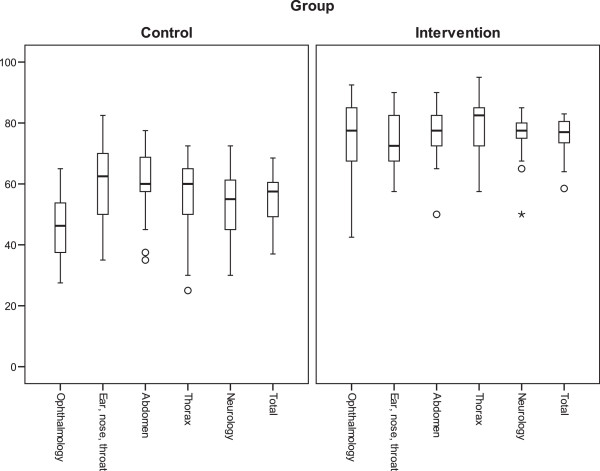
**Percentage of points scored in the five subjects of the OSCE and in total.** Bold lines represent medians, boxes the interquartile ranges, the upper and the lower whiskers the 2.5th and the 97.5th percentile (circles and asterisks are outliers).

Students in the control group scored a median of 57% (25th percentile 47%, 75th percentile 61%) of points possible compared to 77% (73%, 80%; p for difference between groups < 0.001) for students in the intervention group in the final OSCE. The 25th percentile in the intervention group was higher than the highest score in the control group (see Figure 2); only two students in the intervention group achieved a lower score than the best student in the control group.

Students in the intervention group consistently scored better than students in the control group in each of the five OSCE stations. The differences were statistically significant (p < 0.01). Except for ENT, the 25th percentile in the intervention group was higher than the 75th percentile in the control group (Figure 2).

**Figure 3:**
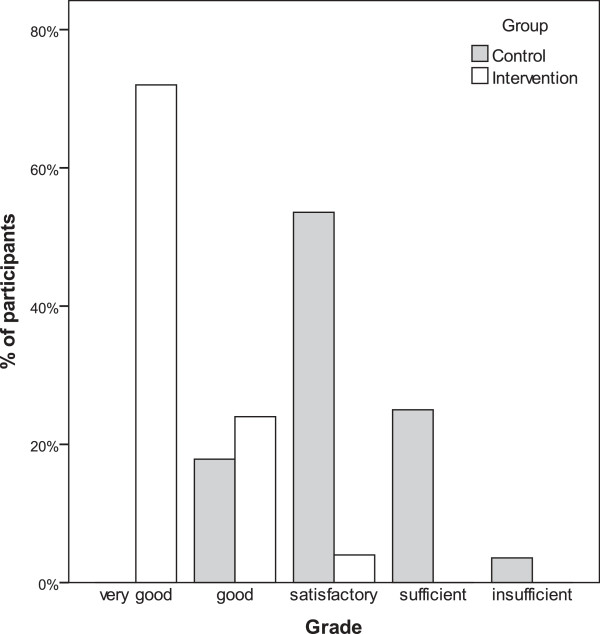
Percentage of participants receiving a given grade in the OSCE.

18 students in the intervention group received the highest grade “very good”, six “good” and one “satisfactory” (Figure 3). In the control group no student received the highest grade, 5 “good”, 15 “satisfactory”, 7 “sufficient” and one did not pass the test (“insufficient”).

In the final meeting, both students and tutors reported that the additional teaching was well accepted by everyone involved. The level of teaching was considered well adjusted to the training level of students. The clinicians stated that the near-pear teaching course had not caused additional time constraints, and that diagnostic and therapeutic measures in daily ward routine had not been disturbed.

## Discussion

Students participating in the additional near-peer teaching course for physical examination scored significantly higher in each subject of an OSCE (ophthalmology, ENT, abdomen, thorax and neurology) compared to students participating only in the routine course. Developing and performing the course proved feasible with limited resources.

It is well known that the learning success of physical examination skills training is dependent to a great extent on interactive small groups and hands-on teaching [7]. However, limited resources often make it challenging for medical schools to implement such an approach in a sufficient manner. We developed our multimodal course in order to minimize the burden for patients and teaching physicians without compromising the effectiveness of didactic techniques. Teaching components proven to be effective in the past were combined in the course. A structured manuscript developed by advanced students has been well received by junior students and was very valuable in a formal structure environment [10]. Peer teaching is a useful concept not only from a practical point of view, but also from the perspective of educational theory [11]. The teaching was done in small student groups of three, led by the near-peer tutors who were fourth or fifth year students [8]. Bedside teaching, one of the best venues for teachers to effectively demonstrate clinical examination techniques [12], occurred under the supervision of the near-peer tutors. To analyse the effectiveness of our approach we used an OSCE which is a reliable and valid tool to assess basic clinical skills [13]. While there is evidence that all these components can improve learning success individually, we are not aware of other randomized studies that evaluated them in combination (Table 1).

Our study has a number of limitations. The most important is that the near-peer course in the intervention group was taught in addition to the mandatory routine course. We would have preferred to compare the near-peer course alone to the routine course alone. However, our near-peer approach initially met considerable scepticism in the medical faculty and we were only allowed to provide it as an addition. Therefore, we cannot claim that our study proves that the new course is better than the old one. Furthermore, we cannot say which aspects of the new course explain the better results in the OSCE. Increase in teaching time, smaller groups, a clearer curriculum, better performance feedback to students, specific training, and close supervision of tutors probably all contribute to improved outcome. Whilst the examiners were blinded as to which teaching the students had, the students were not and the students in the intervention group might have wished to encourage the adoption of the new form of teaching by outperforming the students in the control group and therefore may have actually studied and practised more.

In our near-peer teaching course, students practised clinical examination skills in peers or patients without serious pathology in the area being taught. The aim of the clinical examination course in the third year course is to provide sufficient skills in the examination technique. In the clinical courses following in medical school, students can then adequately train interpretative and clinical diagnostic abilities when examining “real” patients.

Our study did not include a formal process evaluation. There was only an informal feedback meeting after completion of the course, which was attended by all tutors (for administrative reasons) but only a limited number of students. This might have led to an underreporting of criticism. In addition, 37% of participants did not attend the concluding OSCE, as this was voluntary. However, as both groups were similarly affected (33% control group-41% intervention group) there should be a limited effect on the final result. Interestingly, nearly all students had the same excuse for not participating in the final OSCE: the FC Bayern Munich football team was playing at the same time and a large part of students had free tickets for the game. This was not anticipated and became clear when the OSCE started. Unfortunately, there was no formal testing of learning success in the mandatory routine course.

The benefits for near-peer tutors were not evaluated in this study. However, it has been shown previously that student tutors may benefit in several aspects. In order to teach, they have to study clinical skills in more detail, re-learning material to approach it from the perspective of a tutor and not a student. Therefore, they are encouraged to develop new study techniques. The motivation to study for themselves will change and increase. It also prepares them for their future role as teachers, as well as improves leadership skills and self-confidence [5,11,12,14-16].

The experience in our project demonstrated again that advanced medical students can be a tremendous help in curriculum development, as shown in recent research where medical graduates successfully developed a structured curriculum for a near-pear teaching program [10]. To ensure adequacy and broad acceptance of our course, curriculum clinicians, who were responsible for teaching the subject in the routine course, reviewed and corrected the tutorial guidelines when necessary. The structure of the course and rating instruments for the concluding OSCE were developed in a consensus process with clinicians and tutors.

## Conclusion

The findings of our study provide clear evidence that the students who participated to the additional near-pear teaching course scored significantly better on a test which assessed their ability to perform a prescribed physical examination in individuals without severe pathological findings. Based on our experiences and findings, our medical school replaced the former physical examination course by the new near-pear teaching approach although it had been tested as addition to the routine course in our study. The approach integrates several evidence-based components and makes efficient use of limited resources. We believe that our course could be implemented successfully elsewhere.

## Competing interests

The authors declare that they have no competing interests.

## Authors’ contributions

WB designed and managed the study. HB and KL performed the statistical analyses. All authors contributed to the interpretation or the study findings. WB, HB, KL, RV and AS drafted the manuscript. All authors contributed to the revision of the manuscript. All of the authors read and approved the final manuscript.

## Pre-publication history

The pre-publication history for this paper can be accessed here:

http://www.biomedcentral.com/1472-6920/13/165/prepub
